# Clinical Re-Audit of Baseline Assessment, Consideration of Medication, Titration, and Monitoring of Service Users With a Working Diagnosis of ADHD (Attention Deficit Hyperactivity Disorder) in South Durham Tier 3 CAMHS.

**DOI:** 10.1192/bjo.2025.10646

**Published:** 2025-06-20

**Authors:** Mabel Oduma, Thamara Athauda

**Affiliations:** Tees, Esk and Wear Valleys NHS Foundation Trust, Darlington, United Kingdom

## Abstract

**Aims:** This is a re- audit to assess if the same national guidelines in terms of commencement of medications, response to treatment, follow-up, pre/post treatment physical health monitoring, and use of rating scales are being followed for service users with working, but as yet confirmed, diagnoses of ADHD and ascertain utilization of recommendations from previous audit completed in December 2022.

**Methods:** A list of current service users open to South Durham Tier 3 CAMHS with a working diagnosis of ADHD and current trial of stimulant medication was compiled from the team case load.

Required data was collected from the electronic records of identified service users.

**Results:** Previous and current audit had equal sample size of 7 service users with same epidemiological data (sex, age, co-morbidity). All service users had physical and mental health assessment prior to commencing medications in both audits likewise with review of ADHD symptoms recorded following initiation of medication.

In both audits, 5/7 service users had regular contact for follow up, although all of them were offered regular appointments. All eligible service users had physical health check within 3 months of starting a trial of stimulant medication and review of medication side effects.

Like previous audit, 6/7 service users had their height and weight checked and plotted onto a growth chart. As rating scale was utilised to make decisions regarding progress, current audit showed 6/7 completed by parents and school, while none returned by parent and 3/7 schools from previous audit.

Other observations noted include improvement in waiting time from 12 months for majority of service users to 13 to >30 months for all service users from previous audit, attributed to change in assessment team. However, waiting time for initiating medication increased in current audit due to staffing issues.

**Conclusion:** Overall a green compliance was assigned to this clinical audit report, however, some issues were identified in terms of gathering information regarding progress and record keeping. Significant improvement noted following recommendation from previous audit with retrieval of rating scales. Although there is a centralized document with a list of service users as previously recommended, a more detailed document which shows salient information required for follow up will be more helpful. These activities should be added to the agenda of the weekly team meetings to allow monitoring progress in situations of staff sickness.

